# Synthesis and structural analysis of nonstoichiometric ternary fulleride K _1.5_ Ba _0.25_ CsC _60_

**DOI:** 10.3906/kim-2005-78

**Published:** 2020-12-16

**Authors:** Havva Esma OKUR KUTAY

**Affiliations:** 1 Department of Chemistry, Faculty of Engineering and Natural Sciences, Bursa Technical University, Bursa Turkey

**Keywords:** Cation-vacancy, solid-state synthesis, A _6_ C _60_, nonstoichiometric fullerides

## Abstract

The existence of cation-vacancy sites in fullerides might lead to long-range ordering and generate a new vacancy-ordered superstructure. The purpose of this work is to search whether or not long-range ordering of vacant tetrahedral sites, namely superstructure emerges in nonstoichiometric K _1.5_ Ba _0.25_ CsC _60_ fulleride. Therefore, K _1.5_ Ba _0.25_ CsC _60_ with cation-vacancy sites is synthesized using a precursor method to avoid inadequate stoichiometry control and formation of impurity phases within the target composition. For this purpose, first, phase-pure K _6_ C _60_ , Ba _6_ C _60_ and Cs _6_ C _60_ precursors are synthesized. Stoichiometric quantities of these precursors are used for further reaction with C _60_ to afford K _1.5_ Ba _0.25_ CsC _60_ . Rietveld analysis of the high-resolution synchrotron X-ray powder diffraction data of the precursors and K _1.5_ Ba _0.25_ CsC _60_ confirms that K _6_ C _60_ , Ba _6_ C _60_ and Cs _6_ C _60_ are single-phase and they crystallize in a body-centered-cubic structure ( *Im* 3) as reported in the literature. The analysis also shows that K _1.5_ Ba _0.25_ CsC _60_ phase can be perfectly modeled using a face-centered cubic structure. No new peaks appear which could have implied the appearance of a superstructure. This suggests that there is no long-range ordered arrangement of vacant tetrahedral sites in K _1.5_ Ba _0.25_ CsC _60_ .

## 1. Introduction

The intercalated products of fullerides display unique structural, magnetic, and electronic properties which depend on the amount, size, and nature of the intercalated species, and the synthetic route employed for the intercalation of dopant into solid C _60_ , i.e. exohedral doping [1]. Synthetic efforts have been essentially focused on the synthesis of alkali fullerides with stoichiometries A *x* C _60_ (A = alkali metal, 1 ≤ *x* ≤ 12, *e.g.* RbC _60_ , Li _12_ C _60_ ), due to their novel electronic properties. For instance, the observation of metallic behavior in alkali metal intercalated C _60_ films at 300 K [2] was followed by the discovery of superconductivity for the first time in an alkali fulleride K _3_ C _60_ with a transition temperature, *T* c, of 18 K [3], where superconducting phase is a face-centered-cubic (fcc) structure [4,5]. This led to the discovery of new A _3_ C _60_ superconductors through varying the interfullerene separation ( *e.g.* Rb _3_ C _60_ with a *T* c of 28 K [6] and 30 K [4], Rb _2_ CsC _60_ and RbCs2C _60_ with a *T* c of 31 K and 33 K, respectively [7], fcc Cs3C _60_ with a maximum *T* c of 35 K at ~7 kbar [8] and fcc Rb *x* Cs3− *x* C _60_ (0.35 ≤ *x* ≤ 2) with *T* c varying between 25.9 K and 32.9 K at ambient pressure [9]). Since then, extensive research has been carried out on fcc A _3_ C _60_ and A *x* Cs3− *x* C _60_ fullerides to discover superconductors with higher *T* c and understand their molecular electronic structure [8–12]. Current understanding proves that the A _3_ C _60_ superconducting fullerides belong to the family of unconventional superconductivity where electron correlations play an important role for the pairing mechanism [8–11, and 13–18]. Besides that, molecular electronic structure [9,19,20] and cation specific effects [12] are crucially important in producing the unconventional superconductivity in the A _3_ C _60_ family.

Upon exohedral doping of solid C _60_ , the dopant occupies interstitial lattice positions of the solid C _60_ and provides electrons to the host C _60_ molecules, creating C _60_ n- anions [1]. The charge transfer alters the properties of C _60_ (e.g. inducing metallicity and superconductivity) and has thus received the most attention. In the A _3_ C _60_ family, intercalation of three alkali leads to a half-filled *t* 1u band and hence a metallic behavior, excluding fcc Cs _3_ C _60_ [8]. The superconducting phase of the A _3_ C _60_ fullerides adopts the fcc structure where all tetrahedral ( *T* d) and octahedral ( *O* h) holes are entirely occupied by the alkali cations [21]. In fcc A _3_ C _60_ structure, there are two *T* d cavities ( *r* = 1.12 Å) and one *O* h ( *r* = 2.06 Å) per C _60_ unit. As the *T* d cavity is smaller than the *O* h one, the size of the alkali cation occupying the *T* d site instead of the *O* h one will determine the degree of lattice expansion; therefore, the volume of the A _3_ C _60_ fullerides can be changed systematically through substituting the alkali metals with larger ones.

Various synthetic routes have been developed to prepare A *x* C _60_ fullerides. The most common and effective one is the ‘solid-state direct reaction method’ where solid C _60_ is exposed to vapor of the alkali metal which then disperses into the C _60_ acceptor molecules at temperatures of ~100–410 °C. All the A *x* C _60_ and A *x* A’3− *x* C _60_ compounds, except Cs _3_ C _60_ [8,10], can be synthesized with this method [1]. The A _6_ C _60_ fullerides can be synthesized using the direct reaction method and used later as precursors for further reaction with C _60_ to obtain target A *x* Cs3− *x* C _60_ compounds. The precursor method offers a significant benefit compared to the direct synthesis method because fine A _6_ C _60_ powders enable better stoichiometry control of the desired compound. The saturated A _6_ C _60_ compounds can also be prepared by a vapor-transport method [22]. In this study, direct reaction and vapor-transport methods were used to synthesize the K _6_ C _60_ /Ba _6_ C _60_ and Cs _6_ C _60_ precursors, respectively, which were later reacted with C _60_ to afford the target composition.

Besides the exploration of cation related effects on the structural and electronic properties of fullerides, the effects of cation-vacancy can be investigated as well. For example, tetrahedral rare-earth metal vacancies in Sm2.75C _60_ and Yb2.75C _60_ exhibit long-range ordering of tetrahedral vacancies, generating a superstructure [23,24]. Such structural response to tetrahedral vacancy could be established in fcc A *x* Cs3− *x* C _60_ fullerides, resulting in a different structure to the fcc. For such an exploration, in this work, [ ]0.25K _1.5_ Ba _0.25_ CsC _60_ —where [ ] represents vacant tetrahedral sites of the fcc structure — was synthesized using stoichiometric quantities of single phase, fine, black colored K _6_ C _60_ , Ba _6_ C _60_ , and Cs _6_ C _60_ powders together with C _60_ as starting materials. Here, an easy method for the synthesis of nonstoichiometric K _1.5_ Ba _0.25_ CsC _60_ is presented together with the results of Rietveld analysis of high-resolution synchrotron X-ray powder diffraction data collected at ambient conditions for the structural investigation.

## 2. Materials and methods

### 2.1. Synthetic route

All sample operations were performed in an argon-filled glove box (MBraun MB 200B, H2O and O2 < 0.1 ppm) due to the extreme air- and moisture-sensitivity of reactants. As purchased pristine C _60_ (MER corporation, 99.9%) was sublimed prior to synthesis. Solid C _60_ (~500–600 mg) were first ground and then loaded in a quartz ampoule with a separating striction. This was followed by degassing for 3–4 h in a dynamic vacuum of 10−4–10−5 mbar. The sublimation process was undertaken in a tube furnace at 550 °C (ramping 10 °C/min) under dynamic vacuum. As soon as the sublimation ended, the sublimed C _60_ was transferred to the glove box for later use. A Swagelok fitting with J. Young tap (Sigma-Aldrich Corp., St. Louis, MO, USA) was always used while the samples within the tubes were removed or introduced from or into the glove box. Each cycle of annealing of the intermediate and final products was ended through allowing the furnace to cool down to room temperature with a rate of 5 oC/min. Once each annealing cycle was complete, these products were ground using a pestle and mortar to improve crystallinity.

*Synthesis of K* *6* *C* *60*

Stoichiometric amount of K metal was placed in a 5 mm diameter Ta cell and then stoichiometric amount of sublimed C _60_ was introduced onto K in the cell. The cell was then located in a glass tube enclosed with a Swagelok fitting, removed from the glove box, evacuated for 30 min before sealing under 350 mbar of He gas pressure. The sealed sample within the glass tube was placed vertically in a box furnace and heated to 250 °C with a rate of 5 °C/min, followed by another 20 h annealing and cooling to ambient temperature with the same rate as heating. After the initial annealing was completed, the product was transferred to the glove box, removed from the reaction vessel, and ground. The ground product was then pressed into a pellet, introduced into a Ta holder for further period of 2 days annealing at 300 °C to increase the crystallinity.

*Synthesis of Cs* *6* *C* *60*

Phase-pure Cs _6_ C _60_ was synthesized by a vapor transport method using a ~2.3× excess amount of Cs. The solid C _60_ was first placed into a glass tube with a striction. The cesium metal was inserted in a small glass capsule (~7 mm diameter) which was lowered in the glass tube down to where the striction was. The tube enclosed with a Swagelok fitting was removed from the glove box and evacuated for 30 min before sealing under ~400 mbar of He gas pressure. A 3-zone horizontal tube furnace was used in order to create a temperature gradient between Cs and C _60_ for vapor transport. The zone where Cs locates was ramped to 350 °C from room temperature with a rate of 2 °C/min and held there for 3 days. Simultaneously, the zone where C _60_ locates was ramped to 330 °C (2 °C/min) for transportation of vapor from the Cs to C _60_ zone. Before the product was cooled down to ambient temperature, the temperature gradient was reversed to get rid of any undoped Cs from the freshly formed Cs _6_ C _60_ . Once the cooling was complete, the product was removed from the glass tube, ground thoroughly, pelletized, and located in a Ta cell with tightened screw ends which was then sealed under He, after evacuation under dynamic vacuum, and annealed at 350 °C for another 3 days to increase the crystallinity.

*Synthesis of Ba* *6* *C* *60*

Prior to synthesis, in order to obtain oxide-free barium powder, the oxidized surface of a barium rod was first removed by filing. These initial filings were discarded, and a new diamond file was used to exfoliate the required mass of clean fine powder. Once the clean fine powder was obtained, stoichiometric amount of barium powder and C _60_ were mixed and ground using a mortar and pestle, and then pressed into a pellet. The reaction mixture was loaded into a Ta holder which was placed a 15 mm diameter quartz tube then evacuated for 30 min before sealing under 500 mbar of He gas pressure. The sealed mixture was heated inside a muffle furnace using the following thermal protocol: from ambient temperature to 550 °C; held for 1 h; to 650 °C; held for 13 h; to 700 °C; held for 4 h; to 720 °C; held for 18 h. The ramping rate between each step of heating was 5 oC/min. After cooling to room temperature, the product was removed from the reaction vessel, ground, and finally pressed into a pellet and inserted into the same Ta holder for reannealing at 740 °C for 16 h.

*Synthesis of the target compound* K _1.5_ Ba _0.25_ CsC _60_

K _1.5_ Ba _0.25_ CsC _60_ was prepared by a solid state synthetic route according to the following stoichiometric equation describing the ideal reaction: 6K _6_ C _60_ + 4Cs _6_ C _60_ + Ba _6_ C _60_ + 13C _60_ → 24K _1.5_ Ba _0.25_ CsC _60_ . Stoichiometric amounts of K _6_ C _60_ , Ba _6_ C _60_ , Cs _6_ C _60_ , and sublimed C _60_ precursors were mixed and ground thoroughly. The ground mixture was pelletized, introduced into a Ta cell with tightened screw ends which was then placed in a quartz tube, and evacuated for 30 min before sealing under 450 mbar of He gas pressure. The sealed pellet was then positioned in a muffle furnace at room temperature and heated at 600 °C for 16 h with an initial ramp rate of 5 °C / min, finally, the furnace was switched off to cool down to room temperature. The intermediate product was removed from the tube in the glove box, ground, and then pressed into a pellet which was introduced into the same Ta holder for further period of annealing at 300 °C for 1 h and 600 °C for 16 h with the same heating rate with 2 intermediate grindings and pelletizations to increase crystallinity.

### 2.2. Instrumentation

Ambient temperature high-resolution synchrotron x-ray powder diffraction (SXRPD) data of the precursors were collected with the diffractometer on beamline ID31 (λ = 0.40006 Å for K _6_ C _60_ and Cs _6_ C _60_ , and λ = 0.399838 Å for Ba _6_ C _60_ ) at the ESRF, Grenoble, France. SXRPD data of the target compound K _1.5_ Ba _0.25_ CsC _60_ were collected on beamline BL44B2 (λ = 0.500127 Å) at the SPring-8, Japan. The samples were loaded into 0.5 mm diameter glass capillaries and sealed under ~350 mbar He pressure for the SXRPD measurements.

SXRPD data were analyzed using the Rietveld refinement technique with the GSAS suite of the Rietveld programs [25]. The following procedure was applied for the Rietveld analysis: a complex peak shape function known as the pseudo-Voigt, which is a combination by addition of Gaussian and Lorentzian functions [26] was used to model the peak shape, and peak shape coefficients *GU, GV, GW, LX, LY* and *Lij* (i, j = 1–3) were refined. Low-angle peak asymmetry rising from axial divergence was modeled with coefficients *S/L* = 0.001, *H/L* = 0.0005 where *L* is the diffractometer radius, and *H* and *S* are the sample and detectors heights, respectively [25]; a Chebyschev polynomial function (~20 terms) was applied to fit the background; the anomalous contributions to the X-ray form factors of all atoms, f’ and f’’ corrections to f, were calculated (in e/atom) using the program DISPANO [27] and implemented into GSAS as follows: for  λ = 0.4 Åf’ = 0.054, f’’ = 0.079 for K, f’ = −1.771, f’’ = 0.819 for Ba and f’ = −1.921, f’’ = 0.758 for Cs and for λ = 0.5 Å f’ = 0.094, f’’ = 0.125 for K, f’ = −1.190, f’’ = 1.228 for Ba and f’ = −1.247, f’’ = 1.138 for Cs. Intermediate refinements of lattice parameters, occupancies of the tetrahedral sites, thermal parameters, peak shape coefficients, zero correction, and background function were applied during the Rietveld analysis.

## 3. Results

### 3.1. Structural characterization of the K _6_ C _60_ , Cs _6_ C _60_ and Ba _6_ C _60_ precursors

**Figure 1:**
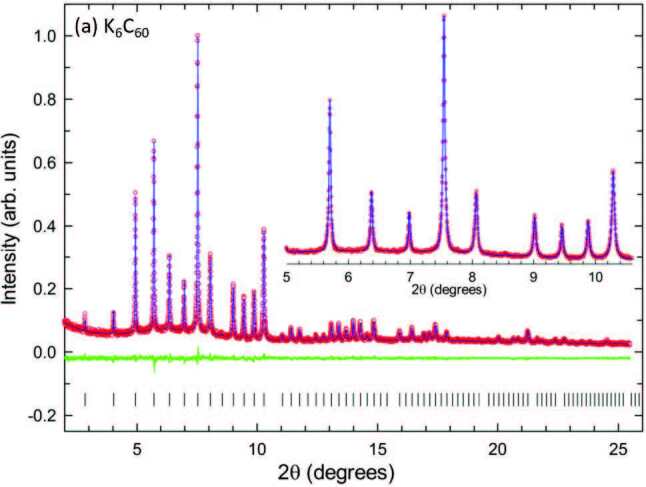

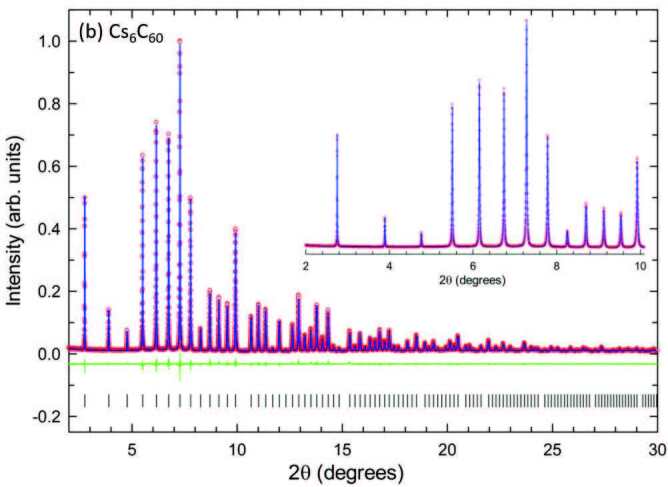

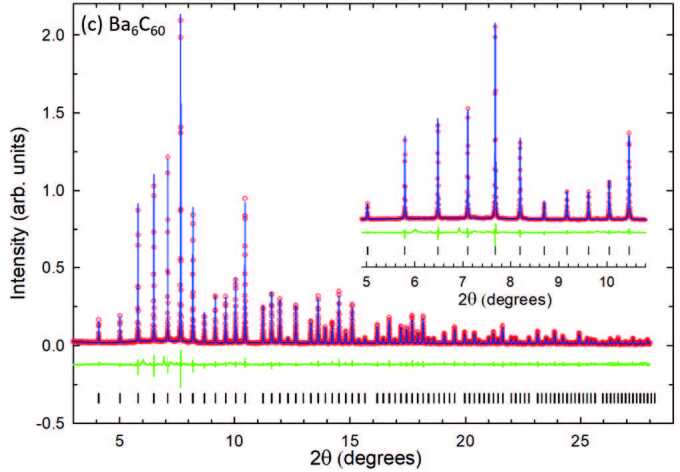
Rietveld fits to synchrotron XRPD data collected at ambient temperature for phase-pure (a) K6C60 (λ = 0.40006 Å, Rwp = 4.43% and Rexp = 4.03%), (b) Cs6C60 (λ = 0.40006 Å, Rwp = 4.23% and Rexp = 3.91%) and (c) Ba6C60 (λ = 0.39984 Å, Rwp = 3.04% and Rexp = 2.20%). Red circles, blue line, and green line represent the observed, calculated, and difference profiles, respectively. Black ticks mark thereflection positions of K6C60, Cs6C60 and Ba6C60 (Im3 ‒ ). Insets show expanded regions of the relevant diffraction profiles.

Rietveld refinements of the SXRPD data (Figure 1) collected for K _6_ C _60_ , Cs _6_ C _60_ , and Ba _6_ C _60_ confirm that samples are high quality, phase-pure, and crystallize with a body-centered-cubic structure with a space group of *Im* 3 ^‒^ and lattice parameters *a* K _6_ C _60_ = 11.3775(2) Å, *a* Cs _6_ C _60_ = 11.7887(2) Å, and *a* Ba _6_ C _60_ = 11.1879(2) Å respectively. These values are in agreement with the previously-reported lattice parameters: 11.39 Å [28], 11.79 Å [29], and 11.1850(7) Å[30], respectively. This confirms that the synthesized A _6_ C _60_ fullerides can be used effectively as precursors. Fractional atomic coordinates of K _6_ C _60_ , Cs _6_ C _60_ , and Ba _6_ C _60_ were taken from [28], [29], and [31], respectively and were not refined. Only lattice constants and thermal displacement parameters, which were modeled isotopically, were refined together with instrumental ( *e.g.* zero shift) and profile shape coefficients. As seen in Figure 1, a good agreement between the calculated and observed profile is obtained from the Rietveld analysis with χ2 ~1.

### 3.2. Structural characterization of the target compound K _1.5_ Ba _0.25_ CsC _60_

**Figure 2:**
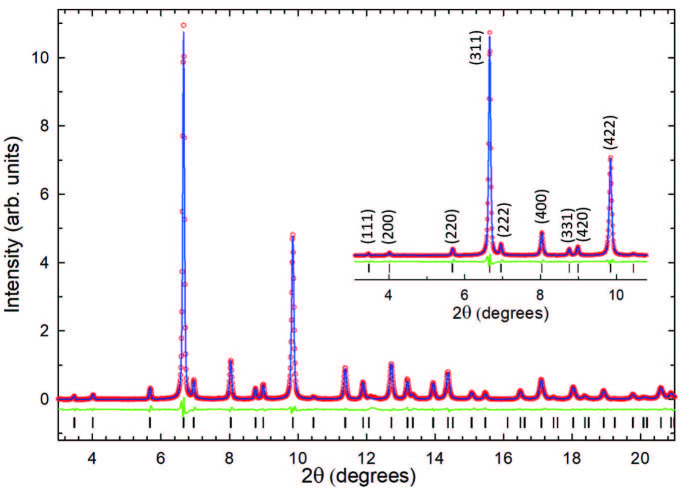
Rietveld fits to synchrotron XRPD data collected at ambient temperature for phase-pure fcc K _1.5_ Ba _0.25_ CsC _60_ (λ = 0.500127 Å, Rwp = 2.76% and Rexp = 1.29%). Red circles, blue line, and green line represent the observed, calculated, and differenceprofiles, respectively. Black ticks mark the reflection positions of fcc phase (space group Fm3 ‒ m). The inset shows an expanded region of the diffraction profile with observed Bragg peaks labeled with their (hkl) Miller indices.

Rietveld analysis of the X-ray diffraction data of K _1.5_ Ba _0.25_ CsC _60_ readily reveals that the sample is single phase and adopts the cubic structure with fcc symmetry (Figure 2). All diffraction peaks can be indexed with the cubic structure, with no sign of additional reﬂections that could have indicated the existence of a vacancy-ordered superstructure. A merohedrally disordered fcc model with the space group of *Fm* 3 ^‒^ *m* was employed to model the fcc phase. A cation disordered model was applied as follows. As the *O* h interstitial site of the fcc C _60_ structure (r = 2.06 Å) is significantly larger than the *T* d one (r = 1.12 Å), larger Cs+ ions preferentially reside in the *O* h site. As a result, the *O* h cavity is only occupied by Cs+ while the smaller K ^+^ and Ba ^2+^ ions (rK ^+^ = 1.38 Å, rBa ^2+^ =1.35 Å, rCs+ = 1.67 Å) preferentially occupy the *T* d site. Hence, the latter is filled by a disordered mixture of K ^+^ and Ba ^2+^ . The applicability of this method has been previously confirmed by 133Cs, 39K, and 87Rb NMR measurements [9,32].

The fractional atomic coordinates of the fcc phase were not allowed to refine, instead they were rescaled from fcc Rb _3_ C _60_ (with C _60_ C‑C bond distances of 1.42 Å [28]). Thermal displacement parameters of the atoms ( *U* ) were modeled isotropically and allowed to refine but under the condition that *U* iso of the C atoms and *U* iso of the K ^+^ and Ba ^2+^ ions introduced into the tetrahedral site were forced to be equivalent to each other, respectively. The K ^+^ and Ba ^2+^ occupancy in the *T* d site of the fcc structure was allowed to refine but total site occupancy was fixed at 1.75, and the remaining is the vacant tetrahedral site occupancy which is 0.25. The refined occupancy ratio converged to K ^+^ : Ba ^2+^ = 0.712(10):0.163(10) leading to a refined composition of K _1.42(1)_ Ba _0.33(1)_ CsC _60_ . The structural parameters of the fcc phase obtained from the Rietveld refinement are summarized in Table.

**Table:** Refined structural parameters for fcc-structured K _1.5_ Ba _0.25_ CsC _60_ (refined composition K _1.42(1)_ Ba _0.33(1)_ CsC _60_ ) from Rietveld analysis of SXRPD data collected at ambient temperature, with λ = 0.500127 Å. Site multiplicities and occupancies are listed in columns M and N, respectively. Values in parentheses are estimated errors from the least-squares fitting. The weighted-profile and expected R-factors are Rwp = 2.76%, and Rexp = 1.29%, respectively. The lattice constant is a = 14.2616(1) Å.

	x/a	y/b	z/c	M	N	Uiso (102 Å2)
K	0.25	0.25	0.25	8	0.712(3)	1.8(1)
Ba	0.25	0.25	0.25	8	0.163(3)	1.8(1)
Cs	0.5	0.5	0.5	4	1.0	7.8(1)
C(1)	0	0.04985	0.24176	96	0.5	0.6(1)
C(2)	0.21102	0.08059	0.09949	192	0.5	0.6(1)
C(3)	0.18008	0.16107	0.04989	192	0.5	0.6(1)

**Figure 3:**
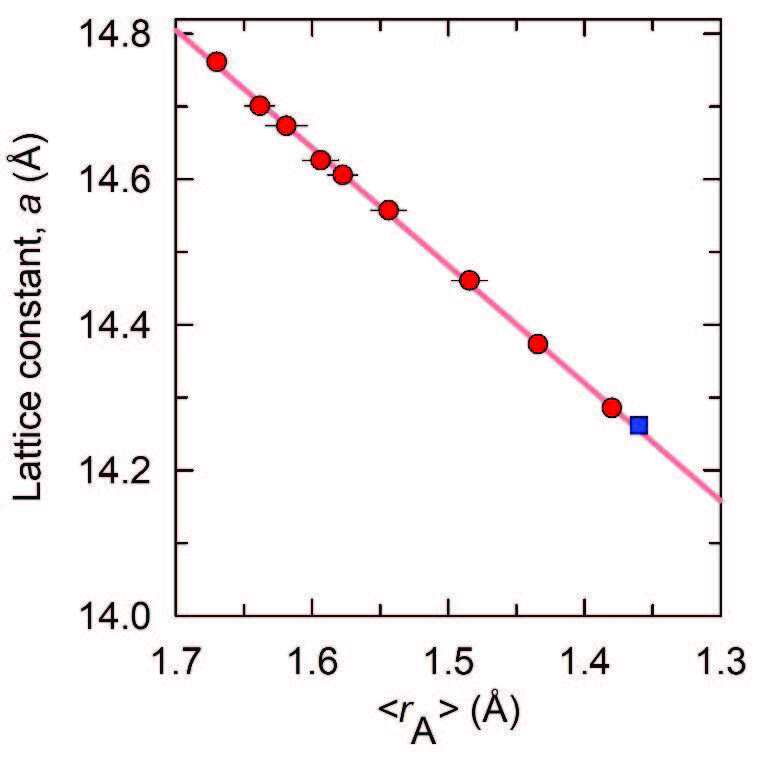
Variation of the ambient temperature fcc lattice constant of K _1.5_ Ba _0.25_ CsC _60_ (blue square) and KxCs3-xC60 (0 ≤ x ≤ 2, red circles, data taken from [32]) with average ionic radii of the cations residing in the Td and Oh cavities, ‹rA›. The solid line through data points is a linear fit, yielding a value of da/d‹rA› = −1.61(1) Å.

Indeed, substitution of smaller Ba ^2+^ for the K ^+^ cation, and the presence of the *T* d vacancy in fcc KxCs3−xC _60_ led to a significant lattice contraction. The fcc lattice parameter of refined composition K _1.42(1)_ Ba _0.33(1)_ CsC _60_ , *a,* is obtained as 14.2616(1) Å, which is smaller than those of any KxCs3−xC _60_ ternary compositions (Figure 3), covering the compositional range 0.22(1) ≤ x ≤ 2 and lattice constants14.28571(7) Å ≤ *a* ≤ 14.7011(2) Å [32]. This reflects the fact that the average ionic radii of the cations residing in the *T* d and *O* h cavities, *‹* *r* *A* › in K _1.42(1)_ Ba _0.33(1)_ CsC _60_ is smaller, *‹* *r* *A* ›= 1.36(1) Å, than that of any KxCs3−xC _60_ compositions (1.38 Å ≤ *‹* *r* *A* › ≤ 1.64(1) Å). Refined isotropic thermal displacement parameters of the cations occupying the *T* d and *O* h cavities, and also of the C atoms are found to be in good agreement with those known from the literature[12]. Because of the smaller size of the *T* d site compared with the *O* h one, thermal displacements of the atoms residing in the former one is expected to be relatively smaller than the ones in the latter.

## 4. Discussion

The molecular, alkali doped A _3_ C _60_ fulleride family possesses remarkable physical properties (e.g. unconventional superconductivity, and strong electron correlations) originating from their molecular electronic structure which can be easily tuned via physical/chemical pressure and temperature without altering their high fcc symmetry. The donor intercalants in molecular fulleride family possessing unique properties are not only limited to alkali and alkaline earth metals, for example, rare-earth doped Yb2.75C _60_ fulleride becomes superconducting below 6 K and shows an exceptional crystal structure contrary to the literature [24]. In a hypothetical Yb3C _60_ , Yb cations reside at the centers of the *O* h and *T* d sites of the fcc C _60_ lattice. However, in Yb2.75C _60_ ,Yb cations occupy off-centered interstitial sites and leave one out of every eight *T* d sites vacant. These vacancy sites display long-range ordering, generating a new cation-vacancy-ordered superstructure, which leads to a unit cell with dimensions twice as large as those of the common fcc fulleride structures [24]. A similar situation is also encountered in Sm2.75C _60_ [23]. In both cases, their complex structure arises from the long-range ordering of tetrahedral rare-earth metal vacancies. In this study, we also aimed to induce such structural response to the presence of vacancy site in K _1.42(1)_ Ba _0.33(1)_ CsC _60_ , however, this could not be achieved. Inspection of the diffraction profile did not reveal any superlattice peaks at low angles that could be indexed to an enlarged unit cell, which may possibly signify the generation of a superstructure as in Sm2.75C _60_ and Yb2.75C _60_ . The underlying physical origin of this type of vacancy-ordering was attributed to a strong directional interaction between electron-poor, charge-deficient five-membered rings of C _60_ and divalent ytterbium cations but not to the cation size difference [24]. However, this suggestion might not be valid in the case of using larger cations as in the present study, i.e. K ^+^ (1.38Å), and Ba ^2+^ (1.35 Å), than that of the tetrahedral hole (1.12Å) but the ionic radius of Yb ^2+^ and Sm ^2+^ are 1.02 and 1.14 Å, respectively, comparable to that of the tetrahedral hole. As it is well known, the size and amount of the dopant species has a primary effect on the crystal structure of fullerides, for instance, contrary to the literature, at low temperatures, the structure of Na _2_ CsC _60_ ( *r* Na+= 1.02 Å) is primitive cubic ( *Pa* 3 ^‒^ ) being isostructural with pristine C _60_ and undergoes a phase transition on heating to an fcc phase with a space group of *Fm* 3 ^‒^ *m* [33], and the structure of Yb2.75C _60_ is orthorhombic with space group *Pcab* [24]. Therefore, it could be tentatively suggested that the size of the cations residing in the tetrahedral site should be taken into account if one aims to generate a long-range arrangement of vacant sites, namely a superstructure.

## 5. Conclusion

In conclusion, the preparation of the nominal K _1.5_ Ba _0.25_ CsC _60_ fulleride using stoichiometric quantities of K _6_ C _60_ , Cs _6_ C _60_ , and Ba _6_ C _60_ precursors, which overcomes inadequate stoichiometry control, via solid-state synthetic route is presented. Structural characterization of the precursors and the target compound was performed with Rietveld analysis of the high-resolution synchrotron X-ray powder diffraction data. The analysis confirmed that the structure of the precursors is body-centered-cubic ( *Im* 3 ^‒^ ) as reported in the literature and free from impurity phases such as oxides of the metals which can be easily formed during the synthetic protocol applied.

The X-ray diffraction pattern of the target compound K _1.5_ Ba _0.25_ CsC _60_ (refined composition: K _1.42(1)_ Ba _0.33(1)_ CsC _60_ ) has shown no evidence for the emergence of superstructure peaks which could have resulted from the long-range ordering of the vacant tetrahedral sites. All the Bragg reflections existing in the diffraction pattern originate from the cubic structure ( *Fm* 3 ^‒^ *m* ) without any violation. This suggests that there is no long-range ordered arrangement of tetrahedral alkaline-earth metal vacancies in K _1.42(1)_ Ba _0.33(1)_ CsC _60_ . The reason for this could be the use of highly symmetric Cs _3_ C _60_ fulleride as a parent phase and of moderately large dopant species, i.e. K ^+^ , Ba ^2+^ . This issue merits more detailed investigation through the synthesis and characterization of vacancy-doped fullerides with lower symmetry, for instance, primitive cubic Na2CsC _60_ could be gradually substituted nonstoichiometrically by a smaller divalent cation which might generate ordering of cation-vacancy and also noninteger valence of C _60_ .
